# H_2_S Sensor Based on Cr_2_O_3_-ɣFe_2_O_3_ Nanoparticles Synthesized via Photolysis Method

**DOI:** 10.1155/tswj/4211483

**Published:** 2024-12-23

**Authors:** Hawraa Kassem Hami, Hussain Ismail Abdulah

**Affiliations:** Department of Chemistry, College of Science, Mustansiriyah University, Baghdad, Iraq

**Keywords:** Cr_2_O_3_-ɣFe_2_O_3_, gas sensor, H_2_S, operating temperature (°C), semiconductor metal oxides

## Abstract

A H_2_S gas sensor has been manufactured using Cr_2_O_3_-ɣFe_2_O_3_ nanoparticles with ratios of (2:1, 1:1, and 1:2), using the photolysis method. The chemical composition and microstructure of the resulting samples were characterized using XRD, EDx, and SEM. The sensor's gas-sensing performance was examined at various operating temperatures ranging from 100°C to 250°C. The results demonstrated that the sensor had optimal response in detecting H_2_S gas at a ratio of 1:2. Furthermore, the discussion revolved around the sensor's sensing mechanism specifically for H_2_S.

## 1. Introduction

Various natural sources contribute to air pollution, such as dust, volcano eruptions, and wildfires [1]. Human activity, whether accidental through the release of chemicals into the environment or intentional through the emission from industrial facilities or various human activities, is primarily responsible for air pollution [2, 3]. According to WHO data, nearly the entire world's population (99%) inhales air that is above the organization's recommended standards and contains high, elevated amounts of contaminants. Furthermore, low- and middle-income nations experience the highest rates of exposure to air pollution [4].

H_2_S is classified as an air contaminant. It is characterized as a colorless gas with a pungent odor and is flammable. Volcanoes, hot springs, and waste decomposition are natural sources of this gas. Additionally, there are several industrial sources, such as oil and gas processing, mining, rayon manufacturing, and biogas production. Detecting H_2_S gas is crucial in environmental monitoring due to its potential to induce detrimental health consequences and even fatalities. Exposure to H_2_S inhibits cytochrome oxidase, prevents oxygen absorption, and can cause death [5, 6].

Gas sensors play an important role in fire prevention, health monitoring, and detecting gas-related risks [7]. Several types of gas sensors exist, including chemical, electrochemical, optical, and capacitive sensors [8]. Gas sensors made of nano-metal oxide semiconductors (NOSs) are the best despite their requirement for heating and affected by humidity. NOS gas sensors are considered the best option due to their high efficiency and ease of use in sensing a wide range of chemicals. They are also characterized by being low cost and working efficiently in industrial environments [9, 10]. A NOS is a fraction of an electronic circuit or a physical phenomenon that may detect changes in a substance's structure caused by harmful chemical reactions [11]. Due to their cost effectiveness, easy manufacturing, and high reliability, NOSs have gained widespread use [12–14]. Various H_2_S gas sensors, such as those made of CuO-SnO_2_ [15], Fe_2_O_3_/MoSe_2_ [16], Fe_2_O_3_/SiO_2_ [17], Fe_2_O_3_/ZnO [18], Pt-Fe_2_O_3_ [19], Cr_2_O_3_-decorated ZnO [20], and Cr_2_O_3_-TiO_2_ [21], have employed variable NOS.

In this article, the photolysis method was used to prepare a NOS Cr_2_O_3_-ɣFe_2_O_3_ NOS in different ratios (2:1, 1:1, 1:2) and use it to detect H_2_S gas. The results showed an increase in the sensitivity of the sensor to detect the gas by increasing the ratio of ɣFe_2_O_3_ in the composite.

## 2. Materials and Methods

### 2.1. Chemicals

Chromium nitrate nine hydrate Cr(NO_3_)_2_·9 H_2_O (≥ 99.0%) and Fe(NO_3_)_3_ • 9 H_2_O (≥ 99.0%) were supplied from Merck, India. Potassium hydroxide (KOH) (≥ 97.0%), PVC, and N-methyl-2-pyrrolidone were supplied from Sigma-Aldrich. Deionized water (DW) was utilized to form all aqueous solutions.

### 2.2. Methods

Pure Cr_2_O_3_ [22], Fe_2_O_3_ [23], and Cr_2_O_3_-Fe_2_O_3_ were prepared using the photolysis method. To synthesize, a solution of Cr(NO_3_)_2_·9 H2O and Fe(NO_3_)_3_·9 H2O in different ratios (2:1, 1:1, 1:2) was mixed and stirred for 30 min. The mixture was then placed in a photolysis cell, which consisted of a quartz tube with a UV light source (125 W, *λ* = 365 nm), inside a Pyrex tube as a reactor, and irradiated for 30 min in an ice bath. A drop of KOH (6 N) is added to form a brown precipitate. The precipitate was washed multiple times with deionized water, separated using a centrifuge at 4000 rpm, dried at 80°C for several days, and then calcined at 600°C for 3 h.

### 2.3. Manufacture of Gas Sensor and Measurement

The gas sensor film was prepared by crushing a mixture of NOS powder with PVC as a binder to obtain a fine powder. Next, we added a solvent of N-methyl-2-pyrrolidone (C_5_H_9_NO) to obtain a smooth paste that is easy to shape on the surface of a slide of glass in dimensions (2 × 3 cm) and placed in an oven at a temperature of 100°C for 4 h to dry the film [24]. A homemade sensor device was used to determine the sensor's response in an operation temperature range of 150°C–250°C for the five sensors, and the H_2_S gas was used at a concentration of 50 ppm. The ratio of change of resistance in the presence and absence of gas is usually expressed in terms of response according to equation (1), where *R*_*a*_ is the air resistance while *R*_*g*_ is the resistance of H_2_S gas [25].

(1) $$\begin{array}{c} \text{Response}=\left(\frac{R_{a}}{R_{g}}\right). \end{array}$$

The response and recovery times refer to the durations required for the sensor to achieve 90% of the overall resistance change. Data were gathered from three gas sensors that were identical and manufactured using the same technique [26].

## 3. Results and Discussion

### 3.1. Structural and Morphological Characteristics

**Figure 1:**
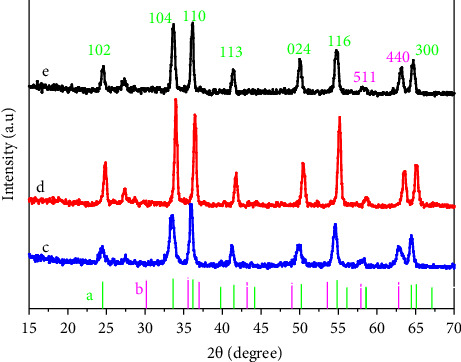
(a) Cr_2_O_3_ (JCPD no. 96-900-8085), (b) ɣFe_2_O_3_ (JCPDS card 39-1346), (c) Cr_2_O_3_-ɣFe_2_O_3_ (2:1), (d) Cr_2_O_3_-ɣFe_2_O_3_ (1:1), (e) Cr_2_O_3_-ɣFe_2_O_3_ (1:2).

Figure 1 clearly illustrates the XRD results for the Cr_2_O_3_-ɣFe_2_O_3_ NOS. There is good agreement compatibility with rhombohedra Cr_2_O_3_ (JCPDS No. 96-900-8085) [27] and ɣFe_2_O_3_ (JCPDS card 39-1346). There was some absence of the expected *γ*Fe_2_O_3_ peaks at 30.26° (220), 35.66° (311), and 43.3° (400), which could be due to peak overlap or suppression by the Cr_2_O_3_ phase [28]. Also, sharp and narrow peaks appear, which confirms that the manufactured material has a good crystalline nature, and the average particle size determined from the Debye–Scherrer formula [14] showed that the increased ratio of Cr_2_O_3_ in the composite led to an increase in particle size. The average particle size for Cr_2_O_3_-ɣFe_2_O_3_ (2:1), (1:1), and (1:2) was 19.21 nm, 16.79 nm, and 13.53 nm, respectively.

**Figure 2:**
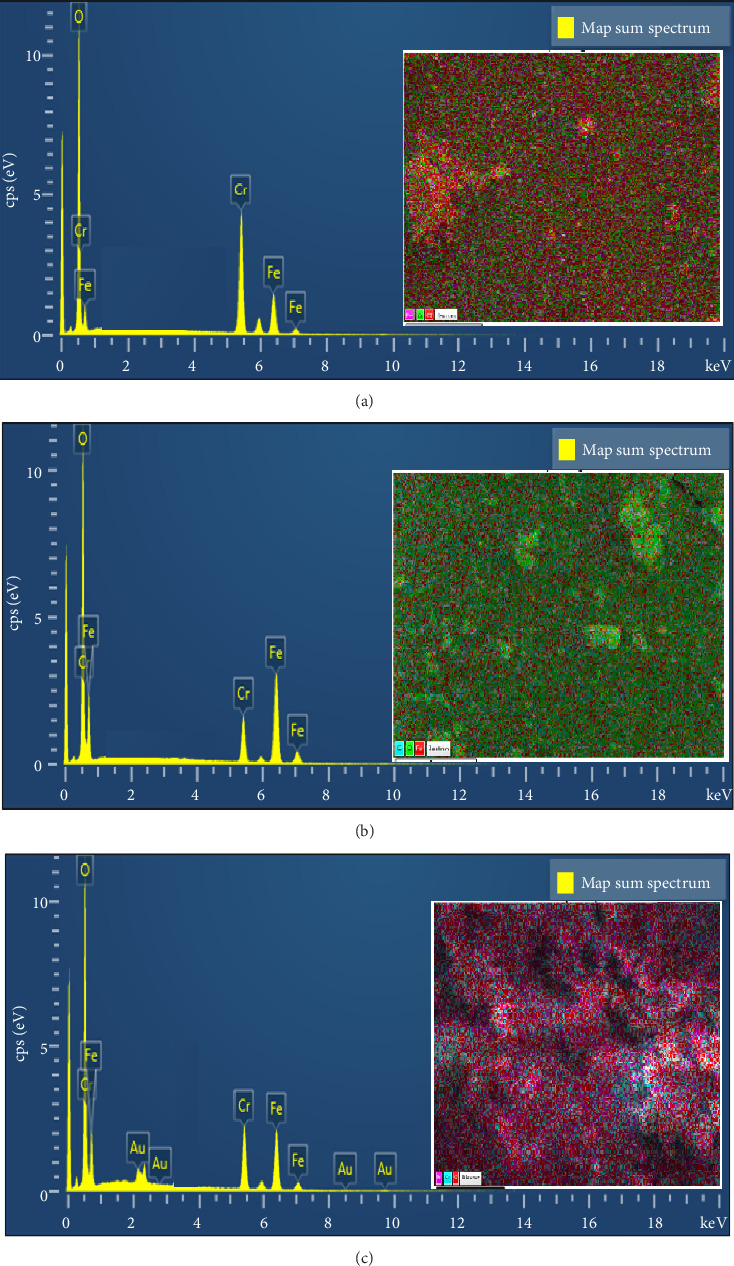
EDX spectra and EDX map of elements in the structure of Cr_2_O_3_-ɣFe_2_O_3_ NOS. (a) (2:1), (b) (1:1), and (c) (1:2).

EDX analysis mentioned the presence of chromium, iron, and oxygen as the main elements (Figure 2), and the results confirm the purity of the prepared nanocomposites. Furthermore, the theoretical calculations of the elements agree with the experimental estimates obtained from EDX. From the EDX mapping analysis in Figure 2, we observe a uniformly dispersion of Cr, Co, and O atom in the composites.

**Figure 3:**
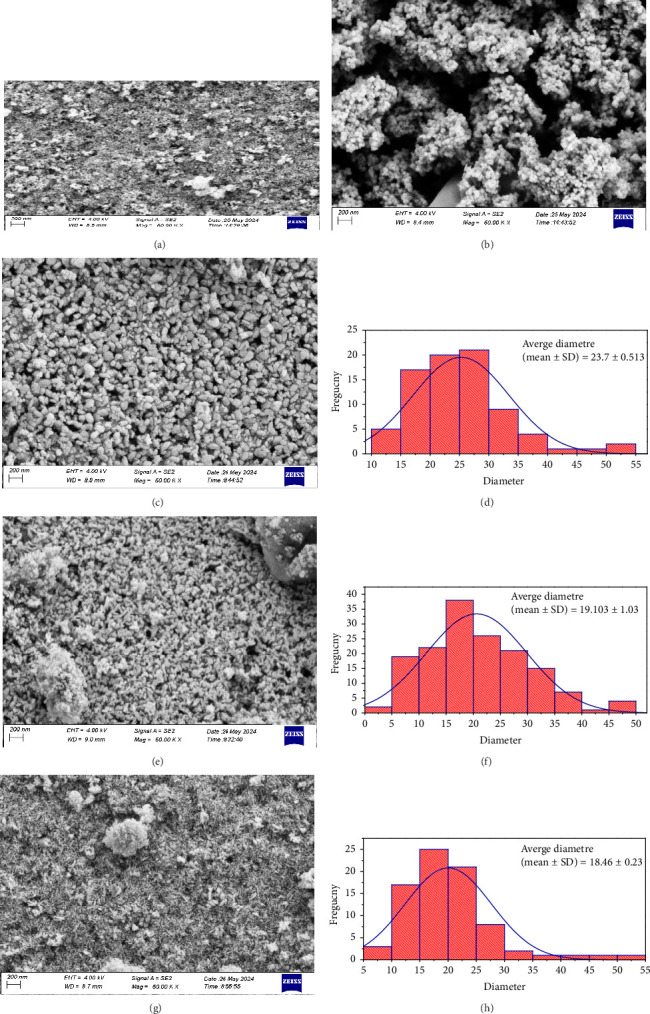
FESEM image of (a) pure ɣFe_2_O_3_ NOS, (b) pure Cr_2_O_3_ NOS, FESEM image and Gaussian distribution of (c, d) Cr_2_O_3_-ɣFe_2_O_3_ NOS (2:1), (e, f) Cr_2_O_3_-ɣFe_2_O_3_ NOS (1:1), and (g, h) Cr_2_O_3_-ɣFe_2_O_3_ NOS (1:2).

In the FESEM technique, an intense beam of electrons is projected onto the sample's surface to be examined. This technique helps in studying the surface morphology and composition of samples [29]. The FESEM images indicate that the pure iron and chromium oxides existed in the form of spherical particles (Figures 3(a) and 3(b)). While the image of the composites Cr_2_O_3_-ɣFe_2_O_3_ NOS in the ratio (2:1, 1:1) shows a random distribution of small-sized granules, ɣFe_2_O_3_ distributed among larger granules Cr_2_O_3_ with the presence of some porous, which can be useful in increasing the sensitivity of the gas sensor (Figures 3(c) and 3(e)) [30]. As for the composite Cr_2_O_3_-ɣFe_2_O_3_ NOS (1:2), the nanoparticles were in the form of cylinders with some spherical clusters. The Gaussian distribution of the grains showed that the average grain size in Figure 3(g) increases with the increase in the percentage of chromium oxide in the composite. A Gaussian fit was used to find the size distribution of all ratios.

## 4. Gas Sensor Properties'

The sensor was manufactured using a mixture of ɣFe_2_O_3_ and Cr_2_O_3_ in different ratios. To study the impact of temperature on the sensor response, the sensors prepared with a concentration of 50 ppm of H_2_S were tested at different operating temperatures ranging from 150°C to 250°C. As evident from the data presented in Table 1, the most favorable temperature for the ratio (2:1, 1:1) was 150°C, which is lower than the optimum operating temperature for pure chromium and iron oxides, which was 200°C, while the ratio (1:2) was at 200°C. At 250°C, there was no noticeable response to H_2_S gas. This could be due to the higher temperature, which reduced the chemical reaction required to sense the gas. Consequently, the resistance remained high.

**Figure 4:**
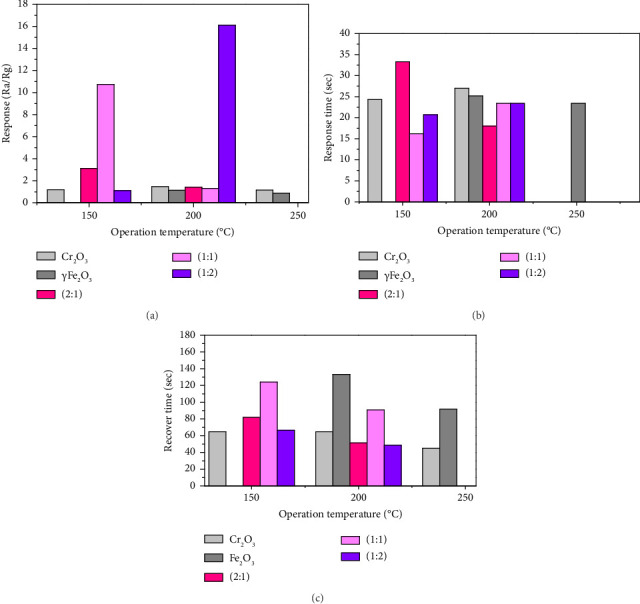
(a) Response of sensor toward gas H_2_S, (b) response time, and (c) recovery time.

The results showed a significant improvement in the sensor's response to H_2_S gas compared to pure Cr_2_O_3_ and ɣFe_2_O_3_ results showed a positive correlation between the sensor response and the ratio of iron in the Cr_2_O_3_-ɣFe_2_O_3_ NOS. The sensor response value increased with the increase in iron oxide, and the Cr_2_O_3_-ɣFe_2_O_3_ NOS with the ratio 1:2 had the highest response (Figure 4(a)). Moreover, the results of the three composite showed a shorter recovery time and response time compared to the pure metal oxide, and the ratio (1:2) having a shorter recovery time and response time compared to the other ratios in the (Figures 4(b) and 4(c)).

**Figure 5:**
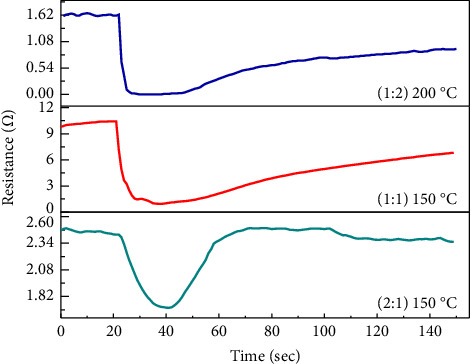
The variation resistance with time at optimum operation temperatures of H_2_S gas for different ratios of Cr_2_O_3-_ɣFe_2_O_3_ NOS.

Figure 5 shows the sensor resistance curve at the optimal temperature for H_2_S gas. The sensor exhibited typical n-type semiconductor behavior, which is attributed to the n-type characteristics of Cr_2_O_3_-ɣFe_2_O_3_ NOS, where the resistance decreased over time in the presence of H_2_S gas and increased over time when the supply was stopped.

**Figure 6:**
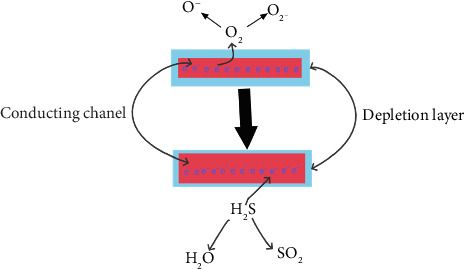
The Cr_2_O_3-_ɣFe_2_O_3_ NOS gas sensing mechanism for sensing H_2_S gas [12].

Figure 6 displays the sensor resistance curve of the optimum temperature for H_2_S gas. The sensor exhibited typical behavior for an n-type semiconductor, which is attributed to the n-type characteristics of Cr_2_O_3_-ɣFe_2_O_3_ NOS, where the resistance decreased over time in the presence of H_2_S gas and increased over time when the supply was stopped [31].

The research results were compared with the results of sensing with sensors containing Cr_2_O_3_ and ɣFe_2_O_3_ NOS in their composition as H_2_S gas detectors, as shown in Table 2.

## 5. Gas Sensing Mechanism

The NOS gas sensor depends mainly on the change in chemical resistance, as the gas interacts with metal oxides as a donor or acceptor to charge carriers, which leads to a change in the resistance or conductivity of the NOS gas sensor [33].

The sensing mechanism of the n-type NOS gas sensor depends on the oxygen molecules adsorbed on the metal surface and forms several forms of oxygen (O_2_^−^, O^−^, and O^2−^) depending on the temperature and material type [12]. As a result, electrons are removed from the conduction band and positive holes are created, creating a negative region on the surface [10]. A stable region is formed as a result of the depletion of electrons due to the separation of charge carriers. In this search, the optimum temperature was between 150°C and 200°C, resulting in the predominant presence of oxygen molecules in the form of O^−^ and a lesser amount of O_2_^−^. When a sensor of the *n*-type is exposed to a reducing gas (H_2_S), the electrons that were previously absorbed by the oxygen are released again. This results in an increase in the concentration of charge carriers and a decrease in the thickness of the electron depletion layer, a corresponding decrease in the resistance of the sensor [34, 35].

## 6. Conclusion

This study synthesized a composite Cr_2_O_3-_ɣFe_2_O_3_ NOS with ratios (2:1), (1:1), and (1:2) using the photolysis method. The results demonstrated that increasing the amount of iron oxide in the composite enhances the sensor's response to detect H_2_S gas. At the optimal operating temperature of 200°C, the ratio (1:2) demonstrated the highest response (16.1) min, and it also had the shortest response and recovery times compared to the other ratios.

## Figures and Tables

**Table 1:** Sensitivity, response time, and recovery time for a pure Cr_2_O_3_, ɣFe_2_O_3_, and Cr_2_O_3-_ɣFe_2_O_3_ NOS membrane at optimum sensing temperature for H_2_S gas.

Material	Optimum *T* (°C)	Response	Response times	Recovery times
Cr_2_O_3_-ɣFe_2_O_3_ (2:1)	150	3.11	33.3	81.9
Cr_2_O_3_-ɣFe_2_O_3_ (1:1)	150	10.73	23.4	144
Cr_2_O_3_-ɣFe_2_O_3_ (1:2)	200	16.1	16.2	48.6
Pure Cr_2_O_3_	200	1.48	23.4	64.8
Pure ɣFe_2_O_3_	200	1.14	24.3	91.8

**Table 2:** Comparison of the gas sensor properties of the present sensor with other sensors containing Cr_2_O_3_ and ɣFe_2_O_3_ NOS in their composition toward the H_2_S gas.

Material	Optimum *T* (°C)	Response	Response times	Recovery times	Ref
Fe_2_O_3_/MoSe_2_	25	57.7	50	53	[16]
Fe_2_O_3_/ZnO	250	5.98			[18]
Cr_2_O_3_-TiO_2_	450	1.22	—	—	[21]
Fe_2_O_3_/NiO	200	8	100	10	[32]
Cr_2_O_3_-ɣFe_2_O_3_ (1:2)	200	16.1	16.2	48.6	This work

## Data Availability

The data that support the findings of this study are available from the corresponding author upon reasonable request.
